# Consumption of L-Proline as Energy Substrate by Cultured Primary Rat Astrocytes

**DOI:** 10.1007/s11064-025-04618-1

**Published:** 2025-11-26

**Authors:** Paul Spellerberg, Ralf Dringen

**Affiliations:** 1 Centre for Biomolecular Interactions Bremen, Faculty 2 (Biology/Chemistry), University of Bremen, P.O. Box 330440, 28334 Bremen, Germany; 2 Centre for Environmental Research and Sustainable Technologies, University of Bremen, Bremen, Germany

**Keywords:** Astrocytes, ATP, Energy, Metabolism, Mitochondria, Proline

## Abstract

The catabolism of the proteinogenic amino acid L-proline in mammalian cells is mediated by mitochondrial enzymes that can oxidize proline to provide energy for mitochondrial ATP regeneration. To investigate the potential of astrocytes to consume and metabolize L-proline, we incubated cultured primary rat astrocytes with L-proline in the absence or the presence of other energy substrates and investigated L-proline consumption, cellular ATP content and cell viability. In the absence of glucose, the cells consumed L-proline which allowed the cells to maintain a high cellular ATP level as long as extracellular L-proline was detectable. This L-proline consumption was saturable and followed apparent Michaelis-Menten kinetics with a calculated K_M_ value of around 320 µM and a V_max_ value of around 100 nmol/(h x mg). In contrast to L-proline, D-proline was not consumed by the cells and was unable to prevent a cellular ATP loss in starved astrocytes. L-Proline consumption was lowered in a concentration-dependent manner by known inhibitors of proline dehydrogenase. The potential of 1 mM L-proline to maintain a high cellular ATP content in starved astrocytes and to prevent cell death was almost identical to that found for 1 mM glucose and a co-application of both substrates had additive ATP-maintaining effects. The presence of L-proline hardly affected the consumption of glucose, while glucose, glucose-derived lactate as well as other energy substrates severely slowed down the astrocytic L-proline consumption. In addition, application of L-proline prevented the rapid loss in cellular ATP level and the subsequent toxicity induced in glucose-deprived astrocytes in the presence of inhibitors of the mitochondrial uptake of pyruvate and fatty acids. These protective effects of proline were abolished by an inhibitor of proline dehydrogenase. The data presented demonstrate that L-proline is an excellent energy substrate for cultured astrocytes especially for conditions of limited availability of other energy substrates.

## Introduction

L-Proline is essential as a proteinogenic amino acid that is needed for protein synthesis [1]. However, L-proline has also been reported to have functions in cellular stress response [2–4], in signaling pathways [5, 6] and as precursor for other amino acids and neurotransmitters [7–9]. In addition, L-proline can serve as an exogenous source of cellular energy generation [6, 10]. Already the initial oxidation of L-proline in mitochondria delivers electrons directly to the respiratory chain, while its further metabolism generates the citric acid intermediate α-ketoglutarate via glutamate [7, 11]. As the complete oxidation of one molecule L-proline fuels the regeneration of up to 34 ATP molecules [11, 12], this amino acid has to be considered as a good energy substrate. Indeed, L-proline has been shown to be extensively used as energy substrate at least in yeast [13], protozoan parasites [14] and flying insects [6, 7].

Due to its special structure as the only secondary amine among the proteinogenic amino acids, L-proline catabolism differs to that of other amino acids [6, 15, 16]. L-Proline oxidation takes place exclusively in the mitochondria and is initiated by proline dehydrogenase (ProDH), also known as proline oxidase, a flavin-dependent enzyme of the inner mitochondrial membrane that is facing the mitochondrial matrix with its active center [7, 9, 17]. ProDH oxidizes L-proline to ∆1-pyrroline-5-carboxylate (P5C) and thereby reduces the ubiquinone of the electron transport chain to ubiquinol [7, 9]. P5C is in a non-enzymatic equilibrium with the non-cyclic glutamate-γ-semialdehyde (GSA) that is further oxidized by P5C dehydrogenase (P5CDH) under reduction of NAD^+^ to glutamate, a precursor of α-ketoglutarate and glutamine. Alternatively, GSA can be metabolized to ornithine by ornithine aminotransferase [7, 9, 18].

For brain slices, it was recently reported that little L-proline metabolism takes place under normo-glycemic conditions, but that the presence of L-proline accelerates mitochondrial metabolism [19]. Although substantial evidence has been provided on the connection of disturbances of L-proline metabolism in the brain with neurological and psychiatric disorders [9, 16, 20, 21], surprisingly little is known on the L-proline metabolism of the different brain cell types. Among the brain cells, astrocytes have a very broad metabolic profile and are considered as key players in the metabolism of the brain. For example, astrocytes have essential functions in the metabolism of glucose, amino acids, neurotransmitters, glutathione and lipids [22–27].

Only a few articles so far have reported some aspects of the astrocytic L-proline metabolism. Cultured astrocytes have been shown to use exogeneous L-proline as precursor for glutamate that is used for the synthesis of glutathione [28]. A high extracellular L-proline level also influences the redox state of cultured astrocytes [29] and increases extracellular glutamate and glutamine concentrations [30]. In addition, the presence of extracellular L-proline increases glutamine synthetase activity and cellular glutamate uptake in astrocytes [30]. Recently, it was found that the consumption of L-proline prevents the loss of cellular ATP in glucose-deprived astrocytes and that this amino acid is the most potent of all proteinogenic amino acids to maintain as an extracellular substrate a high cellular ATP content [10].

In order to investigate the ability of astrocytes to metabolize L-proline in more detail, we exposed cultured primary astrocytes to proline in the absence or the presence of other energy substrates. Here, we show that cultured primary astrocytes consume exogenous L-proline in a concentration-dependent manner and that the presence of other exogenous energy substrates as well as the application of known inhibitors of proline dehydrogenase severely affect astrocytic proline consumption. In addition, the presence of L-proline prevents the loss in cellular ATP levels and the impairment of cell viability that is caused by inhibitors that prevent the mitochondrial uptake of the energy substrates pyruvate and fatty acids. These data demonstrate that L-proline is an excellent energy substrate for astrocytes, especially under conditions of limited availability of other energy substrates.

## Materials and Methods

### Materials and Chemicals

Cell culture materials and transparent and black microtiter plates were purchased from Sarstedt (Nümbrecht, Germany). Dulbecco’s modified Eagles medium (DMEM), fetal calf serum and penicillin G/streptomycin sulfate solution were obtained from Thermo Fisher Scientific (Schwerte, Germany). D-proline, D-glucose, D-fructose, D-mannose, D-β-hydroxybutyrate, L-lactate, ninhydrin and etomoxir were obtained from Sigma Aldrich (Steinheim, Germany). 4-(2-Hydroxyethyl) piperazine-1-ethanesulfonic acid (HEPES) was purchased from Carl Roth (Karlsruhe, Germany) and glacial acetic acid was purchased from Fluka (Buchs, Switzerland) and Sigma Aldrich (Steinheim, Germany). L-Proline was purchased from Fluka (Buchs, Switzerland). UK5099 and pyruvate were obtained from Merck (Darmstadt, Germany). Acetate, NAD^+^, NADH, NADP^+^, perchloric acid, dimethyl sulfoxide (DMSO) and bovine serum albumin (BSA) were purchased from AppliChem (Darmstadt, Germany). Hexokinase, glutamate pyruvate transaminase, lactate dehydrogenase (LDH), glucose-6-phosphate dehydrogenase and adenosine 5’-triphosphate (ATP) were obtained from Roche Diagnostics (Mannheim, Germany). (S)-(-)-Tetrahydro-2-furoric acid (THFA) was from Santa Cruz Biotechnology (Dallas, USA), (S)-(+)−5-oxotetrahydrofuran-2-carboxylic acid (5-Oxo) was from Tokyo Chemical Industry (Tokyo, Japan) and N-propargylglycine (N-PPG) was from MedChem Express (Monmouth Junction, USA). The Cell Titer Glo^®^ 2.0 Assay Kit used for the ATP quantification was obtained from Promega (Walldorf, Germany).

### Astrocyte Cultures

Primary astrocyte-rich cultures were prepared from the total brains of newborn Wistar rats as previously described in detail [31]. The rats were purchased from Charles River Laboratories (Sulzfeld, Germany) and were treated in accordance to the State of Bremen, German and European animal welfare acts. Harvested cells were suspended in culture medium (90% DMEM (containing 25 mM glucose, 44.6 mM sodium bicarbonate, 1 mM pyruvate, 20 U/mL penicillin G, 20 µg/mL streptomycin sulfate) and 10% fetal calf serum) to a density of 300,000 cells/mL. One mL of the cell suspension was seeded into the wells of 24-well plates. The cultures were incubated at 37 °C in a humidified atmosphere containing 10% CO_2_ in a Sanyo (Osaka, Japan) CO_2_-incubator. The culture medium was renewed every seventh day and one day prior to experiments. Experiments were conducted on confluent cultures of an age between 14 and 28 days. Primary cultures of rat astrocytes are strongly enriched for astrocytes and contain only low numbers of other glia cell types [31–33].

### Experimental Incubation of the Cultures

For experiments, the cultured cells were washed twice with 1 mL glucose-free, warm (37 °C) incubation buffer (IB; 145 mM NaCl, 20 mM HEPES, 5.4 mM KCl, 1.8 mM CaCl_2_, 1 mM MgCl_2_ and 0.8 mM Na_2_HPO_4_; the pH was adjusted to 7.4 with NaOH at 37 °C) and subsequently incubated at 37 °C in 250 µL IB containing the indicated substrates and compounds in the humidified atmosphere of a cell incubator without CO_2_ supply. Etomoxir, UK5099, 5-Oxo and N-PPG were dissolved as concentrated stock solutions in DMSO. Therefore, the experiments with these inhibitors included the appropriate solvent control with the final DMSO concentration that never exceeded 1% (v/v). After the respective incubation period, the media were collected for determination of extracellular concentrations of proline, glucose and lactate as well as of the extracellular LDH activity. The cells were washed twice with 1 mL ice-cold (4 °C) phosphate-buffered saline (PBS; 10 mM potassium phosphate buffer pH 7.4, containing 150 mM NaCl) and lysed with ice-cold 0.5 M perchloric acid for the quantification of the cellular ATP content.

### Determination of the Extracellular Concentrations of Proline, Glucose and Lactate

The L-proline concentration of medium samples was determined as recently described [10], using an adaptation of a colorimetric assay [34] which is based on the formation of a ninhydrin-proline product under acidic conditions and at high temperature. D-Proline was quantified by the proline assay [10] with the difference that D-proline was used to generate a suitable calibration curve to quantify D-proline. Extracellular concentrations of glucose and lactate were determined by enzymatic assays as previously described [31]. The glucose quantification is based on the phosphorylation of glucose to glucose-6-phosphate by hexokinase and the subsequent oxidation to 6-phosphogluconate by glucose-6-phosphate dehydrogenase. This indicator reaction generates NADPH from NADP^+^ in equimolar amounts to the initial glucose concentration and can be quantified by an increase in absorbance at 340 nm [31]. Extracellular lactate was quantified by monitoring its oxidation to pyruvate by LDH followed by transamination by glutamate-pyruvate transaminase. The NADH generated by the LDH reaction was quantified via its absorbance at 340 nm and used to calculate the lactate concentration [31].

### Quantification of Cellular ATP Content

For the quantification of the cellular ATP content, cells were lysed for 10 min in 200 µL ice-cold 0.5 M HClO_4_. ATP contents were determined in neutralized lysates as previously described [35] with the Cell Titer Glo^®^ 2.0 ATP assay kit that uses for ATP quantification the bioluminescent reaction of luciferase, converting luciferin to oxyluciferin.

### Determination of Cell Viability and Protein Content

The potential toxicity of a given treatment was assessed as previously described [31] by measuring the extracellular activity of the cytosolic enzyme LDH that had been released from the cell during the treatment due to impaired membrane integrity. The extracellular LDH activity is given in percent as a relative value compared to the initial cellular LDH activity that had been determined by total lysis of the cells with 1% (w/v) Triton X-100 [31]. The protein content of astrocyte cultures was determined by the Lowry method [36] with BSA as standard protein.

### Data Presentation and Statistical Analysis

The data shown represent means ± standard deviation (SD) of values obtained in three or more independent experiments each performed in duplicate or triplicate (for initial values) wells of one culture, if not indicated otherwise. In bar plots and the table, the data obtained in the three independent experiments are also given. Statistical analysis of normal distribution for *n* = 3 data sets are underpowered and not suitable [37]. Therefore, we have tested for normal distribution of data by visual assessment in quantile-quantile plots with a 95% confidence interval. The values determined for individual experiments always followed apparent normal distribution. Analysis of statistical significance of differences between groups of data was performed by one-way Analysis of Variance (ANOVA) or Analysis of Co-Variance (ANCOVA) followed by the Bonferroni post-hoc test. For significant differences found by the ANCOVA analysis, effects sizes were calculated as partial η^2^ values, with η^2^ > 0.14 indicating large effect sizes [38]. For post-hoc comparison of significant differences found by ANCOVA between individual data groups, Cohen´s d values (d) were calculated with d > 0.8 indicating large effect sizes [39]. Analysis of statistical significance of difference between two data sets was performed by a paired, two-tailed t-test. The significance of differences compared to the respective control is indicated by ^#/^*^/+^*p* < 0.05; ^##/^**^/++^*p* < 0.01 and ^###/^***^/+++^*p* < 0.001. *p* > 0.05 was considered as not significant (ns).

## Results

### Consumption of L-Proline by Astrocytes in the Absence or the Presence of Glucose

**Fig. 1:**
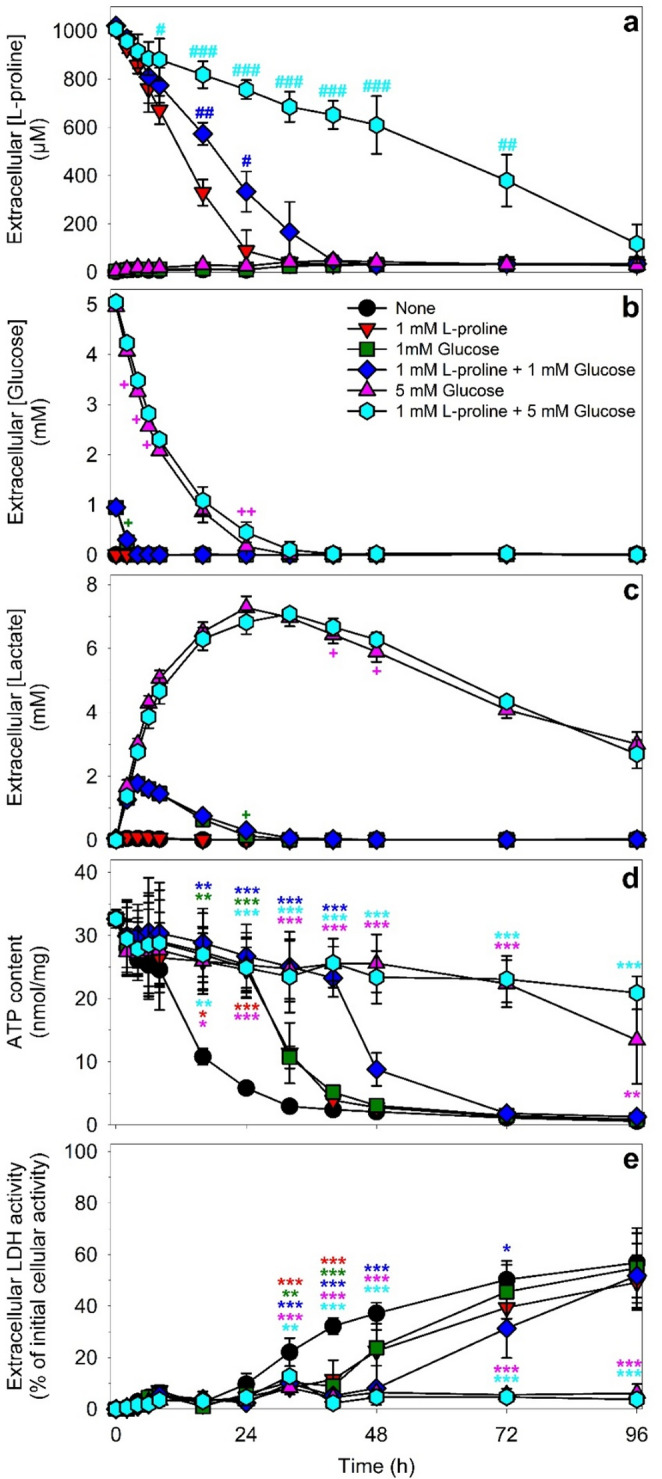
Consequences of an incubation of cultured primary astrocytes without or with L-proline and/or glucose. Primary astrocytes were exposed to glucose-free IB that contained no energy substrate (None) or either L-proline (1 mM) or glucose (1 mM or 5 mM) or combinations of L-proline plus glucose and incubated for up to 96 h. For the indicated time points, the extracellular concentrations of L-proline (**a**), glucose (**b**) and lactate (**c**) as well as the specific cellular ATP contents (**d**) and the extracellular LDH activities (**e**) were determined. In panels b and c, the measured values of extracellular glucose or lactate of the conditions containing initially 1 mM glucose are very similar and the values for the glucose-only condition are thereby hardly visible behind the data points for the respective glucose plus proline condition. The initial cellular LDH activity of the cultures was 147 ± 26 nmol/(min x well) and the initial protein content was 136 ± 14 µg/well. The data represent means ± SD of values obtained in three independent experiments that were performed on independently prepared cultures. Statistical significance of differences (ANOVA with Bonferroni post hoc test) is indicated for the comparison with the L-proline-only condition (**a**) and for comparison with the none condition (**d**, **e**). In panels b and c, the significance of difference (t-test) is indicated for the comparison of data from the respective two conditions that contained an identical initial glucose concentration. Significance levels are indicated by ^#/+/^*p < 0.05; ^##/++/^**p < 0.01 and ^###/+++/^***p < 0.001

**Fig. 2:**
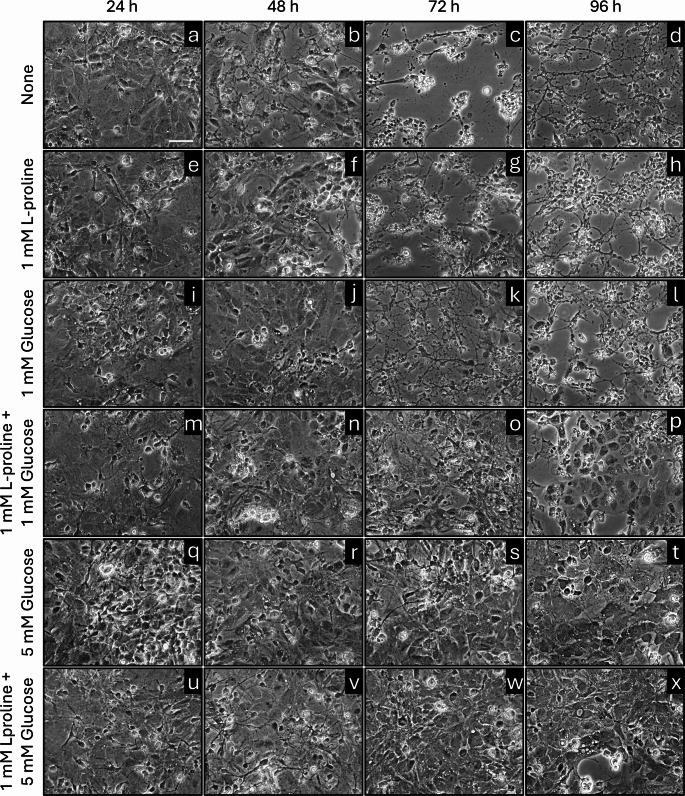
Phase contrast images showing alterations in cell morphology during incubation of cultured primary astrocytes without or with L-proline and/or glucose. The incubation conditions were identical to those described in Fig. 1. Shown are representative images from one experiment that has been reproduced twice on independently prepared cultures with similar results. The scale bar in panel a represents 50 μm and applies to all panels

The presence of exogenous L-proline enabled glucose-deprived astrocytes to maintain a high cellular ATP content for 24 h [10]. In order to study L-proline consumption by primary astrocytes in more detail as well as to elucidate potential interferences between glucose and L-proline consumption, cultured astrocytes were incubated without or with 1 mM L-proline in the absence or the presence of 1 mM or 5 mM glucose for up to 4 d before the extracellular concentrations of L-proline, glucose and lactate, the specific cellular ATP content as well as the cell viability were determined. In the absence of proline and glucose, the specific cellular ATP content of the cultures declined during the first 24 h of incubation to around 20% of the initial content (Fig. 1d), while the cell viability was not compromised as demonstrated by the absence of extracellular LDH activity (Fig. 1e), by the normal cell morphology and by the absence of any obvious loss in cells from the confluent cell layer (Fig. 2a). However, longer incubations of astrocyte cultures without energy substrates lowered the cellular ATP content further (Fig. 1d) and caused severe cell toxicity as demonstrated by the strongly increased extracellular LDH activity (Fig. 1e) as well as by the loss in confluency and the altered morphology of the cultures that showed condensed and damaged cells as well as vesicular structures within some cells (Fig. 2b-d).

After application of 1 mM L-proline, the extracellular L-proline concentration declined almost proportional with time and the applied L-proline was almost completely consumed by the cells during the first 24 h of incubation (Fig. 1a), while at best minute amounts of extracellular glucose (Fig. 1b) and lactate (Fig. 1c) were determined. For astrocytes that had been exposed to 1 mM L-proline, the cellular specific ATP content remained high for 24 h before it declined during longer incubations, reaching around 12% of the initial ATP content within 40 h (Fig. 1d). During longer incubations, the ATP content declined further and the cell viability was compromised (Figs. 1d and e and 2g and h).

After application of 1 mM glucose as exclusive substrate, the applied glucose was fully consumed within 4 h (Fig. 1b) and had been mainly converted to lactate that was released from the cells during this incubation period (Fig. 1c). However, during longer incubations the glucose-derived lactate was consumed by the cells and was not detectable anymore after 24 h (Fig. 1c). During the first 24 h of incubation after application of 1 mM glucose the cellular ATP content remained high, but was lowered within the next 16 h of incubation to around 15% of the initial value (Fig. 1d). During longer incubations, cell viability was compromised as demonstrated by an increase in extracellular LDH activity (after 48 h) (Fig. 1e) and by damaged cells in the microscopical image of the cultures (after 72 h) (Fig. 2k, l).

For 1 mM concentrations of either glucose or L-proline, an almost identical decline in cellular ATP content was observed (Fig. 1d) after the substrates L-proline (Fig. 1a) and glucose plus the glucose-derived lactate (Fig. 1b, c) had been consumed. LDH release (Fig. 1e) and loss in confluency of the cell layer (Fig. 2g, k) were found after the ATP content had substantially declined (Fig. 1d).

After application of 5 mM glucose to astrocytes, the extracellular glucose was almost completely consumed within 24 h (Fig. 1b) and had been mainly converted to lactate (Fig. 1c) that was consumed by the cells during longer incubations. For this condition, the cellular ATP content remained high at least during the first 3 d of incubation (Fig. 1d) and the cell viability was not compromised during incubations for up to 4 d (Figs. 1e and 2t).

Co-application experiments with L-proline plus glucose revealed that the cellular consumption of L-proline by astrocytes (Fig. 1a) was slowed by the presence of either glucose (Fig. 1b) or glucose-derived lactate (Fig. 1c), while the consumption of glucose (Fig. 1b) and glucose-derived lactate (Fig. 1c) was hardly affected by the presence of L-proline. The remaining extracellular concentrations of L-proline determined after 24 h of incubation were 89 ± 85 µM (no initial glucose), 334 ± 83 µM (1 mM glucose) and 757 ± 39 µM (5 mM glucose). The specific rates of L-proline consumption for the three proline-containing conditions investigated, as calculated from the linear decline of extracellular L-proline concentrations during the first 24 h, were 70 ± 14 nmol/(h x mg) (no initial glucose), 52 ± 14 nmol/(h x mg) (1 mM glucose) and 19 ± 4 nmol/(h x mg) (5 mM glucose). In addition, the co-application of 1 mM L-proline plus 1 mM glucose delayed the loss of cellular ATP (Fig. 1d) and the onset of toxicity in the cells (Fig. 1e). For the time range studied, neither the specific cellular ATP content (Fig. 1d) nor the cell viability or culture confluency (Figs. 1e and 2x) were significantly affected during co-application of 1 mM L-proline with 5 mM glucose.

In cell-free experiments performed under otherwise identical conditions to those used for cell experiments, the initial concentration of L-proline (1 mM) was not lowered during incubations at 37 °C for up to 24 h (data not shown), demonstrating that the decline in detectable L-proline in cell experiments was not caused by cell-independent decomposition of L-proline but rather by cellular consumption.

### Consumption of Exogenous L-Proline by Glucose-Deprived Astrocytes

In order to investigate the concentration- and time-dependency of the L-proline consumption, cultured astrocytes were incubated in glucose-free IB containing initial L-proline concentrations ranging from 0 µM to 2 mM for up to 24 h before the extracellular L-proline concentrations were measured and the specific L-proline consumption rates were determined. In the absence of L-proline, a rather low unspecific absorbance from ninhydrin-complexes formed by unknown cell-derived compounds was determined by the proline assay for incubations that were longer than 4 h (data not shown). As these complexes revealed spectral characteristics different from that of the proline-ninhydrin complex and accounted for an apparent L-proline concentration of at best 13 µM (data not shown), we subtracted the absorbance values derived from the unknown cell-derived compounds from the respective total absorbance signals determined for the media of L-proline-treated cells to obtain the real L-proline concentration present for each time point and condition.

**Fig. 3:**
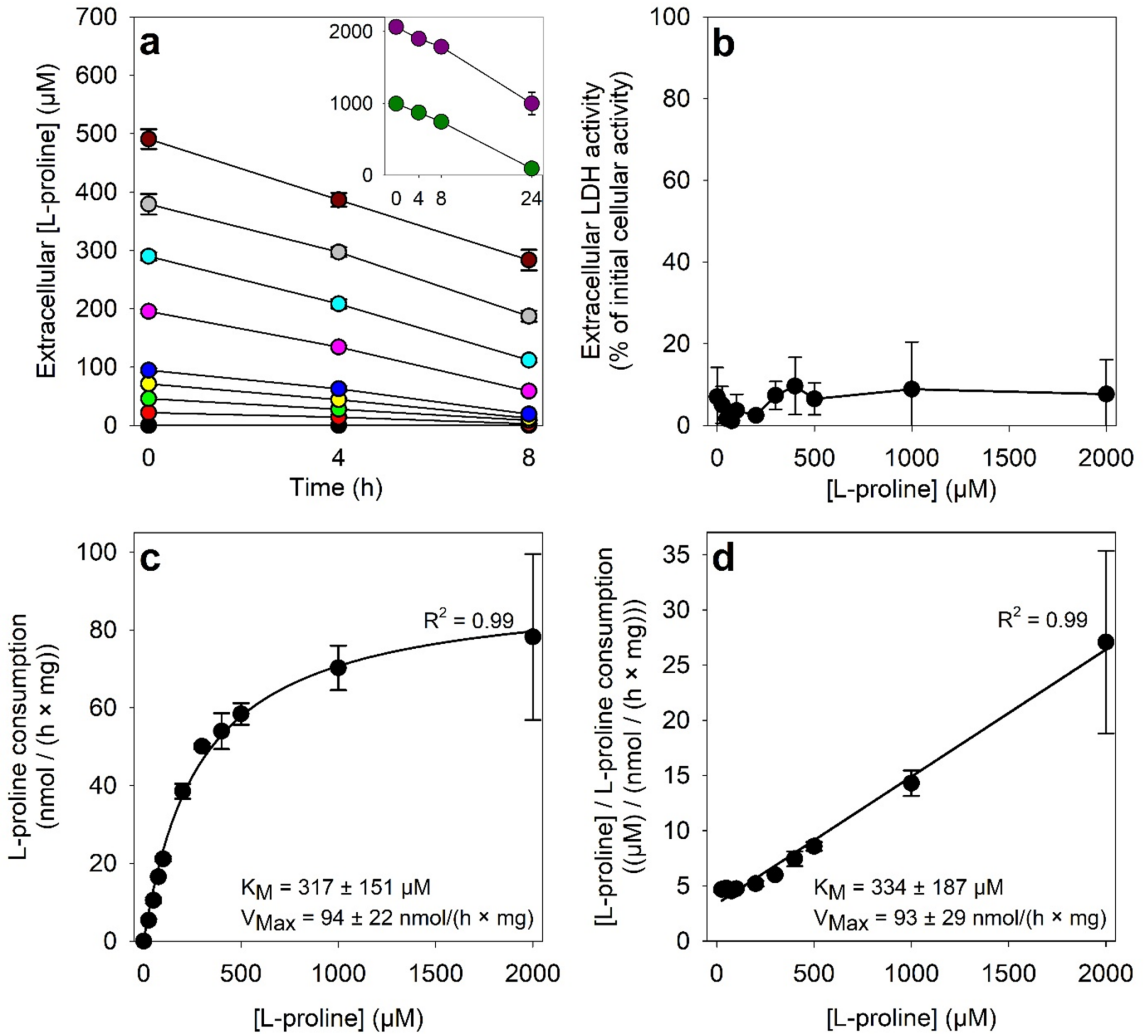
Concentration- and time-dependent L-proline consumption by glucose-deprived astrocytes. The cells were exposed in glucose-free IB to the indicated initial L-proline concentrations for up to 8 h (initial L-proline concentrations of 25 µM − 200 µM) or up to 24 h (no proline and initial L-proline concentrations of 300 – 2000 µM). For the indicated incubation periods the extracellular L-proline concentration was determined and for the respective longest incubation period also the extracellular LDH activity. Panel a shows the extracellular L-proline concentration for the respective time points. The given values had been corrected for the low amount of unspecific signal from an unknown cell-derived compound that was determined for the 0 µM L-proline incubation condition. The linear declines in extracellular L-proline concentrations during the initial 8 h of incubation were used to calculate the specific L-proline consumption rates (c). The linearization of these data was done according to Hanes-Woolf and the respective graph is shown as panel d. Panel b gives the extracellular LDH activity in % of the initial cellular LDH activity. The initial cellular LDH activity of the cultures was 126 ± 7 nmol/(min x well) and the initial protein content was 111 ± 4 µg/well. The data represent means ± SD of values obtained in three independent experiments performed on independently prepared cultures

For each initial concentration of L-proline applied, the cells consumed the amino acid in a time-dependent linear manner (Fig. 3a) that was used to calculate the initial specific L-proline consumption rates. Cell viability was not impaired during the incubations as demonstrated by the low extracellular LDH activity (Fig. 3b). Correlation of the L-proline consumption rates to the initial L-proline concentrations applied revealed an apparent Michaelis-Menten type relationship for the L-proline consumption by cultured astrocytes (Fig. 3c). Calculation of the kinetic parameters of L-proline consumption by fitting of the data with the hyperbolic Michaelis-Menten equation (Fig. 3c) or by analysis of the data after linearization by the Hanes-Woolf equation (Fig. 3d) revealed similar results for L-proline consumption with a K_M_ value of around 320 µM and a maximal L-proline consumption velocity of around 100 nmol/(h x mg protein) (Fig. 3c, d).

**Table 1:** Comparison of the utilization of L-proline and D-proline by cultured astrocytes. The cells were incubated in glucose-free IB that had been supplemented with 1000 µM of either L-proline or D-proline for 24 h, before the concentrations of remaining extracellular proline, the cellular ATP contents and the extracellular LDH activities were determined. The initial specific cellular ATP content was 24.3 ± 2.6 nmol/mg, the initial cellular LDH activity was 163 ± 13 nmol/(min x well) and the initial protein content was 153 ± 11 µg/well. The data represent means ± SD of values obtained in three independent experiments. The values obtained from the three individual independent experiments are given in brackets. Statistical significance of differences (t-test) between the data obtained for the two proline stereoisomers and the respective initial values at the onset of the incubation is indicated by *p < 0.05; **p < 0.01. Statistical significance of differences (t-test) between the data obtained for incubations with the two proline stereoisomers is indicated by ^#^*p* < 0.05 and ^##^*p* < 0.01

Parameter	L-Proline	D-Proline
Remaining [proline] (µM)	217 ± 212* (70, 120, 460)	989 ± 24^#^ (965, 990, 1013)
ATP content (nmol/mg)	20.9 ± 1.9 (18.8, 21.7, 22.3)	5.3 ± 0.6**^, ##^ (4.7, 5.4, 5.8)
Extracellular LDH activity (% of initial)	5 ± 2 (4, 4, 7)	8 ± 6 (2, 11, 11)

For further studies on the consumption of L-proline by cultured astrocytes, either 1 mM L-proline was applied for 24 h or 200 µM L-proline were applied for 8 h. During 24 h incubations with 1 mM L-proline, the extracellular L-proline concentrations were lowered to average values of 199 ± 196 µM, corresponding to an average specific L-proline consumption rate of 59 ± 17 nmol/(h x mg) (mean values ± SD of data from a total of 41 experiments performed on 28 independently prepared cultures). During 8 h incubations with 200 µM L-proline, the extracellular L-proline concentrations were lowered to average values of 61 ± 19 µM, corresponding to an average specific L-proline consumption rate of 33 ± 6 nmol/(h x mg) (mean values ± SD of data from a total of 22 experiments performed on 12 independently prepared cultures). In contrast to L-proline, its stereoisomer D-proline was not consumed by cultured astrocytes and was unable to prevent the cellular loss of ATP in starved astrocytes (Table 1).

### Exogenous Energy Substrates Lower Astrocytic L-Proline Consumption

**Fig. 4:**
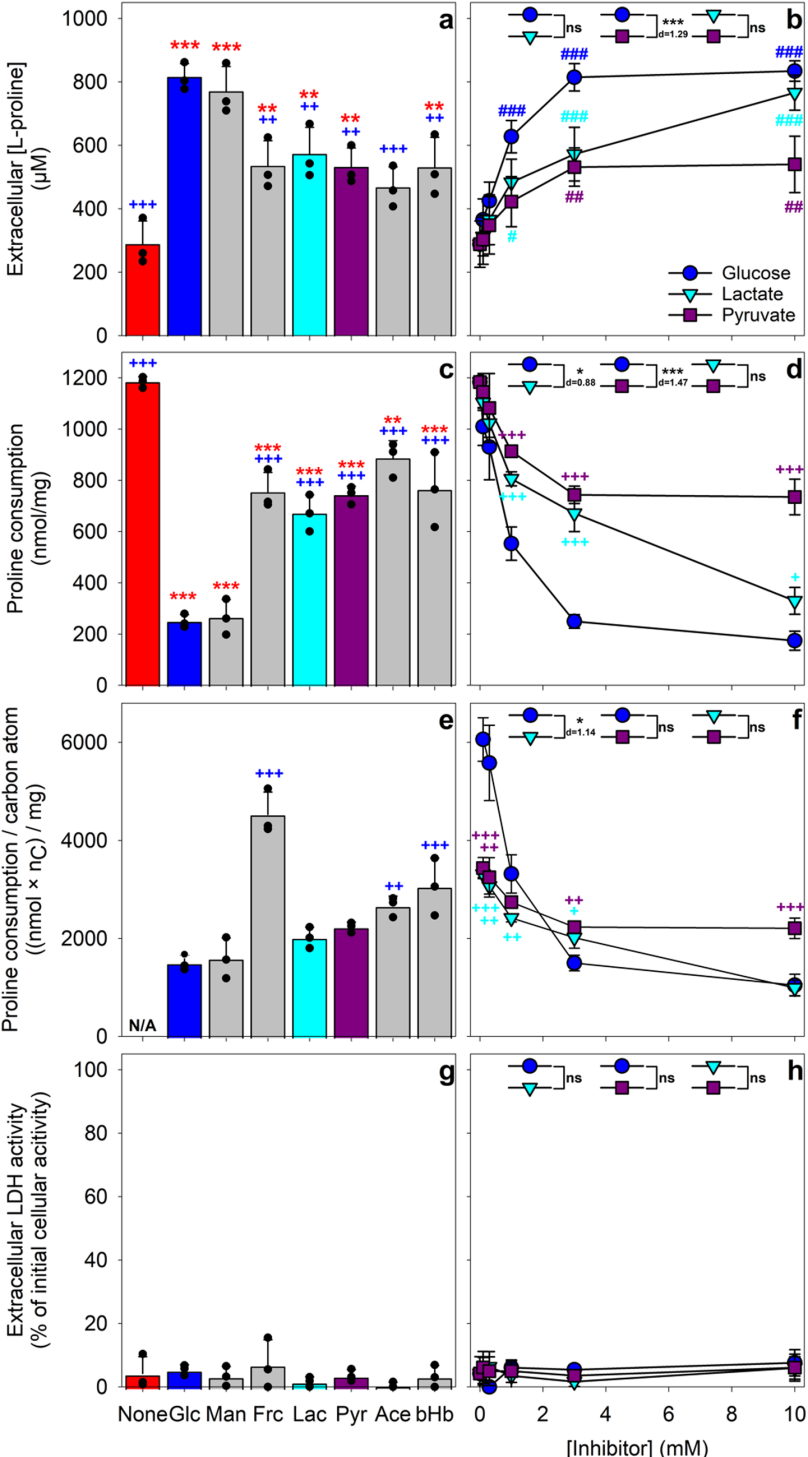
Effects of exogenous energy substrates on the L-proline consumption by cultured astrocytes. The cultures were incubated with 1000 µM L-proline in the absence or the presence of 3 mM of the indicated energy substrates glucose (Glc), mannose (Man), fructose (Frc), lactate (Lac), pyruvate (Pyr), acetate (Ace) or β-hydroxybutyrate (bHB) for 24 h (panels a, c, e and g) or with 1000 µM L-proline in the presence of the indicated concentrations of glucose, lactate or pyruvate (panels b, d, f and h), before the remaining extracellular L-proline concentrations (a, b) and the extracellular LDH activities (g, h) were measured. The specific L-proline consumption during 24 h was calculated for each condition for the applied molar concentration of the other substrates (c, d). In addition, the proline consumption per carbon atom present in the applied other substrates was calculated (e, f). The initial cellular LDH activity of the cultures was 137 ± 16 nmol/(min x well) and the initial protein content was 139 ± 7 µg/well. The data represent means ± SD of values obtained in three independent experiments. Statistical significance of differences (ANOVA with Bonferroni post hoc test) is indicated for the comparison with the control condition (None, asterisks; panels a, c and e), for comparison with the glucose treatment (plus symbols; panels a, c-f) or for comparison with the values obtained for the absence of any inhibitor (0 mM, hashes; panel b) by ^+/^*^/#^*p* < 0.05; ^++/^**^/##^*p* < 0.01 and ^+++/^***^/###^*p* < 0.001. Analysis of Co-Variance (ANCOVA; concentration as co-variable) between the data obtained for the three concentration-dependencies (panels b, d, f, h) revealed a significant impact of the respective substrate inhibitor on the remaining extracellular L-proline concentration (panel b; *p* = 0.001, F(2,50) = 7.6, η^2^ = 0.23), the calculated L-proline consumption (panel d; *p* = 0.0003, F(2,50) = 9.8, η^2^ = 0.28) and the calculated L-proline consumption per carbon atom (panel f; *p* = 0.012, F(2,41) = 5, η^2^ = 0.19). Significance of differences between the three conditions (ANCOVA with Bonferroni post-hoc test) is indicated in panels b, d, f and h with *p < 0.05 and ***p < 0.001 (ns = not significant). For significant differences between two conditions, the effect size (Cohen’s d value) has been included as number below the symbol indicating the level of significance in panels b, d, and f

The L-proline consumption by astrocytes was lowered by the presence of glucose or of glucose-derived lactate (Fig. 1). In order to test for the potential of other exogenous energy substrates to modify the L-proline consumption by primary astrocytes, the cells were incubated for 24 h with 1 mM L-proline in the presence of 3 mM of one of the well-known astrocytic energy substrates glucose, mannose, fructose, lactate, pyruvate, acetate, or β-hydroxybutyrate [10, 35]. The presence of 3 mM glucose significantly reduced the astrocytic L-proline consumption (Fig. 4a) and the specific consumption of exogenous L-proline by around 80% (Fig. 4c). Of the exogenous substrates tested, only mannose had a similarly strong inhibitory effect on L-proline consumption as glucose (Fig. 4a, c). The other exogenous substrates significantly lowered the consumption of exogenous L-proline by cultured astrocytes, but their inhibitory effect was significantly lower than that observed for glucose or mannose (Fig. 4a, c). However, taking the different number of carbon atoms of the investigated substrates into account by calculating the specific proline consumption per carbon atom, revealed that mannose, lactate and pyruvate did not differ significantly from glucose in their potential to slow down proline consumption by astrocytes (Fig. 4e). None of the conditions applied caused any toxicity as concluded from the absence of any significant increase in extracellular LDH activity (Fig. 4g).

The potential of glucose, lactate and pyruvate to lower the L-proline consumption in cultured astrocytes was studied in more detail by co-incubation of astrocytes with 1 mM L-proline plus one inhibitory substrate in concentrations ranging from 0 to 10 mM for 24 h. All three substrates caused a concentration-dependent inhibition of L-proline consumption (Fig. 4b, d). Already the presence of 0.1 mM glucose, 0.3 mM lactate and 1 mM pyruvate inhibited the L-proline consumption significantly, but higher concentrations of these compounds had a stronger inhibitory potential (Fig. 4d). For glucose and pyruvate, an increase in the initial concentration above 3 mM did not further increase their inhibitory potential, while the application of 10 mM lactate lowered L-proline consumption more strongly than 3 mM lactate (Fig. 4b, d). The overall inhibitory potential of glucose on L-proline consumption by astrocytes was significantly higher than that of lactate (*p* = 0.03, d = 0.88) or pyruvate (*p* = 0.0002, d = 1.47) (Fig. 4d). At a concentration of 10 mM, glucose reduced the specific proline consumption by 85%, lactate by 72% and pyruvate by 38% (Fig. 4b, d). However, at concentrations below 1 mM the potential of pyruvate and lactate to slow down proline consumption appears to be as good as that of glucose (Fig. 4d). This becomes more obvious from calculations of the specific proline consumption per carbon atom of the other substrate which revealed that pyruvate and lactate had at concentrations below 1 mM an even stronger potential to slow down proline consumption than glucose (Fig. 4f). None of the conditions applied caused any toxicity as concluded from the absence of a significant increase in extracellular LDH activity (Fig. 4f).

### L-Proline Consumption is Inhibited by Different Inhibitors of Proline Dehydrogenase

**Fig. 5:**
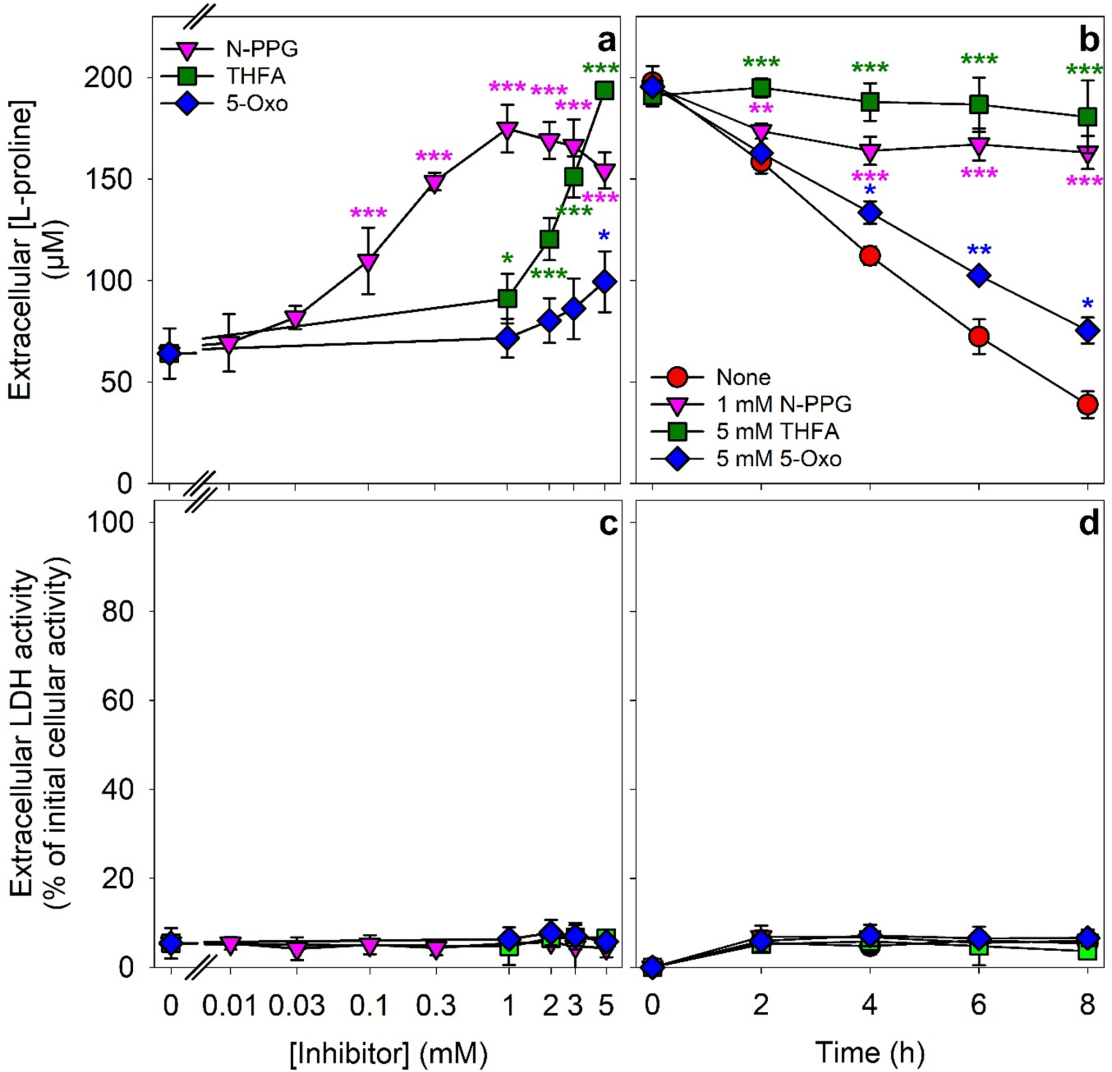
Concentration- and time-dependent inhibition of the L-proline consumption by inhibitors of proline dehydrogenase. Cultured astrocytes were incubated with 200 µM L-proline for 8 h in the absence or the presence of the indicated concentrations of N-PPG, THFA or 5-Oxo (a, c) or with 200 µM L-proline for up to 8 h in the absence or the presence of 1 mM N-PPG, 5 mM THFA or 5 mM 5-Oxo (b, d) before the extracellular L-proline concentrations (a, b) and the extracellular LDH activities (c, d) were determined. The initial cellular LDH activities were 146 ± 7 nmol/(min x well) (a, c) and 170 ± 5 nmol/(min x well) (b, d). The initial protein contents were 139 ± 3 µg/well (a, c) and 138 ± 9 µg/well (b, d). The data represent means ± SD of values obtained in three experiments performed on independently prepared cultures. Statistical significance of differences (ANOVA with Bonferroni post hoc test) of the values obtained for the none condition (absence of inhibitors) with those determined for the given inhibitor conditions are indicated as *p < 0.05; **p < 0.01 and ***p < 0.001

Proline dehydrogenase (ProDH) oxidizes L-proline to P5C and thereby initiates mitochondrial L-proline catabolism [7, 9, 17]. Several compounds have been described as ProDH inhibitors, including (S)-(-)-tetrahydro-2-furoric acid (THFA) [40, 41], S-5-oxo-2-tetrahydrofurancarboxylic acid (5-Oxo) [42] and N-propargylglycin (N-PPG) [42–44]. To test for the potential of these three compounds to inhibit L-proline consumption by cultured astrocytes, the cells were incubated for up to 8 h with 200 µM L-proline in the absence or the presence of various concentrations of the inhibitors (Fig. 5). Of the inhibitors tested, N-PPG and THFA inhibited the consumption of L-proline strongly by more than 80% and 95% in concentrations of 1 mM and 5 mM, respectively, while 5-Oxo even in a concentration of 5 mM had little inhibitory potential on the L-proline consumption by astrocytes (Fig. 5a). Half-maximal inhibition of L-proline consumption was observed for concentrations of around 0.2 mM N-PPG and 2.5 mM THFA. (Fig. 5a). The presence of the different ProDH inhibitors in the concentrations applied did not cause a significant decline in the specific cellular ATP content during the 8 h incubation compared to the absence of the inhibitors (data not shown). During the incubation of astrocytes in the absence or the presence of 5-Oxo, extracellular L-proline concentrations decreased in an almost linear manner over time, but the velocity of the L-proline consumption was slightly reduced by 5-Oxo (Fig. 5b). In the presence of 5 mM THFA, the extracellular L-proline concentration did not decrease significantly (Fig. 5b), while in the presence of 1 mM N-PPG the extracellular L-proline concentration declined slightly during the first 4 h of the incubation, after which the extracellular L-proline concentration remained unchanged (Fig. 5b). During the 8 h incubation with the different inhibitors, the extracellular LDH activity was not significantly increased (Fig. 5c, d). For further studies on the consequences of an inhibition of ProDH, only N-PPG was used as it has been described as an irreversible inhibitor of the enzyme [42–45] and as it was highly effective to lower L-proline consumption in much lower concentrations than THFA (Fig. 5a).

### L-Proline Fuels ATP Maintenance and Prevents Toxicity in Astrocytes in the Presence of Inhibitors of Mitochondrial Pyruvate and Fatty Acid Uptake

**Fig. 6:**
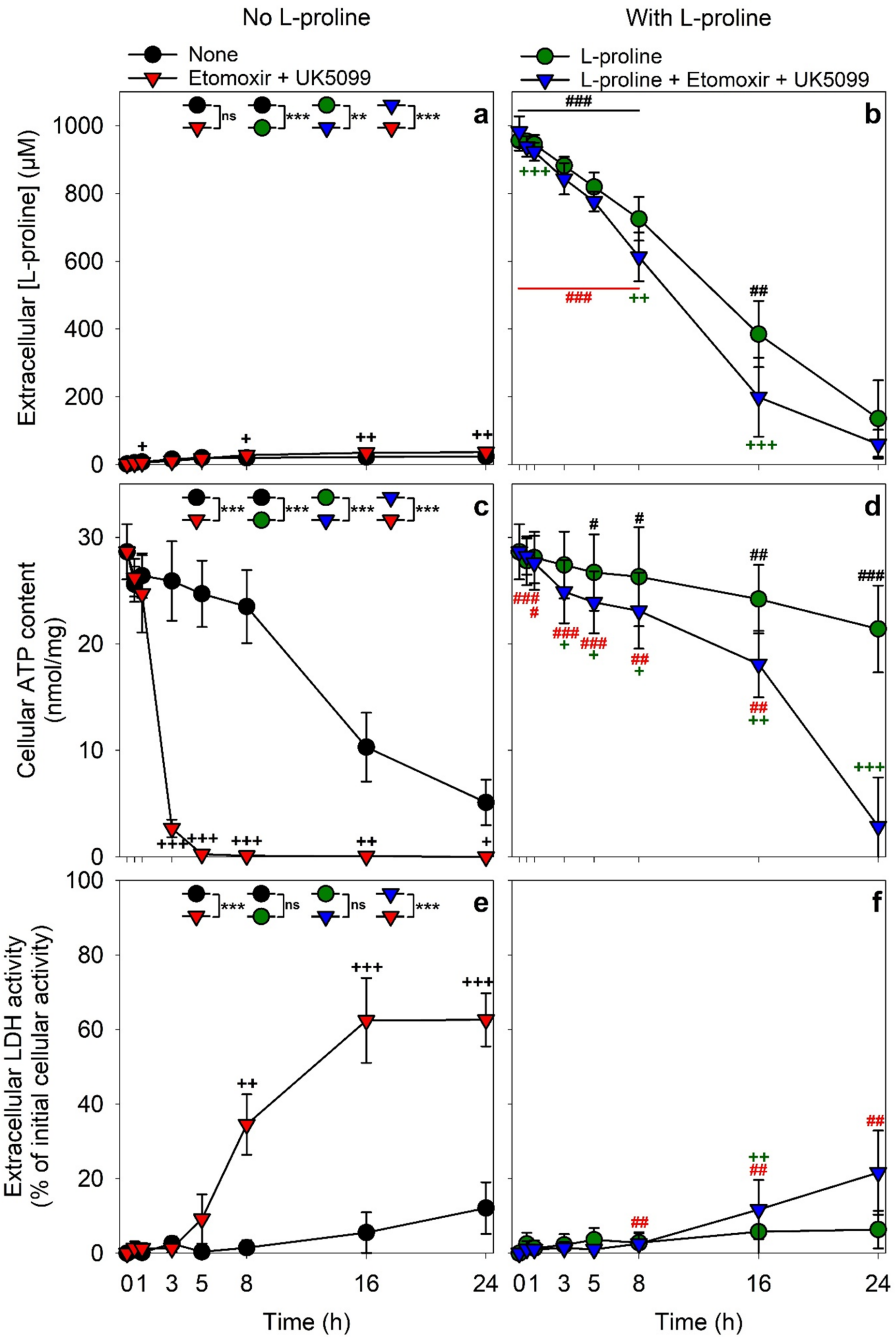
Protective effects of L-proline against the loss in cellular ATP content and cell viability during incubation with etomoxir plus UK5099. Cultured astrocytes were incubated in the absence (a, d, e) or the presence (b, d, f) of 1000 µM L-proline without or with 10 µM etomoxir plus 1 µM UK5099 (inhibitors of the mitochondrial uptake of pyruvate and activated fatty acids) for up to 24 h. At the indicated time points, the extracellular L-proline concentrations (a, b), the specific cellular ATP contents (c, d) and the extracellular LDH activities (e, f) were measured. The initial cellular LDH activity of the cultures was 153 ± 18 nmol/(min x well) and the initial protein content was 147 ± 12 µg/well. The data represent means ± SD of values obtained in four independent experiments performed on independently prepared cultures. Statistical significance of differences (t-test) between either the conditions with or without etomoxir plus UK5099 at the respective time points or between the conditions with or without L-proline at the respective time points are indicated as ^+/#^*p* < 0.05; ^++/##^*p* < 0.01 and ^+++/###^*p* < 0.001. Statistical significance of differences between the treatments were calculated via analysis of co-variance (ANCOVA) with time as co-variable and the treatment as independent variable. Results of the ANCOVAs between two conditions are indicated as **p < 0.01 and ***p < 0.001. Analysis of Co-Variance (ANCOVA; time as co-variable) between the two conditions containing L-proline in the presence or the absence of etomoxir and UK5099 revealed a significant impact of these mitochondrial inhibitors on the decline of the extracellular L-proline concentration (*p* = 0.0012; F(1,61) = 11.588, η^2^ = 0.16) and on the calculated L-proline consumption (*p* = 4.6 × 10^− 7^; F(1,61) = 31.8, η^2^ = 0.34)

During incubation of starved astrocytes without L-proline (Fig. 6a), a high ATP content was maintained during incubations for up to 8 h (Fig. 6c). Longer incubation for up to 24 h caused a gradual decline in cellular ATP content to around 20% of the initial content (Fig. 6c), while the cells remained viable (Fig. 6e). In contrast, in the presence of etomoxir (an inhibitor of the carnitine palmitoyltransferase-1 [46]) plus UK5099 (an inhibitor of the mitochondrial pyruvate carrier [47]), the ATP content of starved astrocytes declined almost completely within 3 h (Fig. 6c) and severe cell toxicity was found during longer incubations with the inhibitors as demonstrated by the strong increase in extracellular LDH activity (Fig. 6e), consistent with literature data [10].

During incubations of astrocytes without etomoxir and UK5099 but in the presence of 1 mM L-proline, the applied L-proline was consumed (Fig. 6b) and most of the initial ATP content was still detectable in the cells after 24 h of incubation (Fig. 6d) in the viable cells (Fig. 6f). For astrocytes that had been incubated with etomoxir plus UK5099 in the presence of L-proline, the applied amino acid was consumed more rapidly by the cells (Fig. 6b), the inhibitor-mediated rapid decline of the cellular ATP content (Fig. 6c) was strongly slowed (Fig. 6d) as long as extracellular L-proline was detectable (Fig. 6b) and the inhibitor-induced severe loss of cell viability (Fig. 6e) was almost completely prevented (Fig. 6f).

**Fig. 7:**
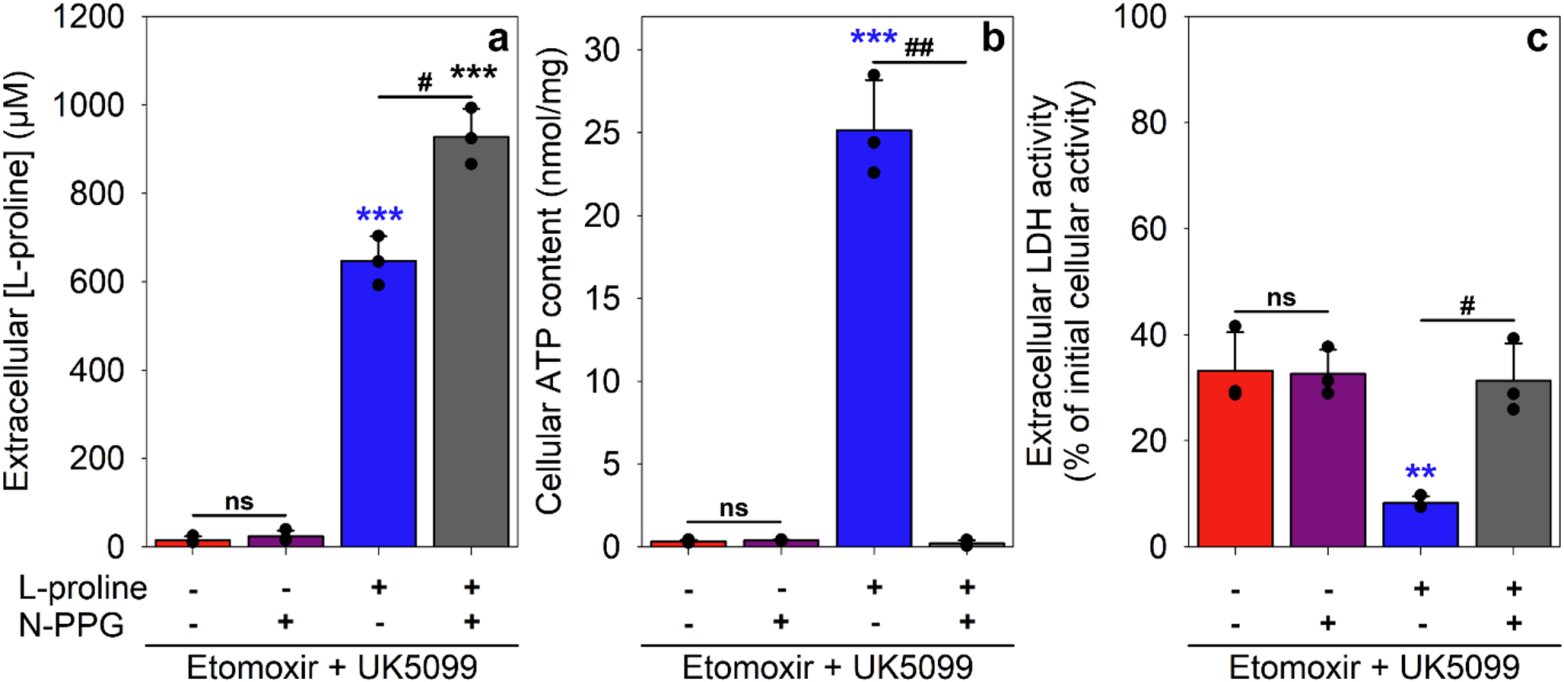
The ProDH inhibitor N-PPG prevents the protective effect of L-proline on starved astrocytes during incubation with etomoxir plus UK5099. The cells were incubated with 10 µM etomoxir plus 1 µM UK5099 without or with 1000 µM L-proline and/or 1 mM N-PPG for 8 h before the extracellular L-proline concentrations (**a**), the cellular ATP contents (**b**) and the extracellular LDH activities (**c**) were measured. The initial cellular LDH activity was 152 ± 10 nmol/(min x well), the initial cellular ATP content was 35 ± 2 nmol/mg and the initial protein content was 138 ± 2 µg/well. Statistical significance of differences between the conditions with the identical initial L-proline concentration (hashes, t-test) or in comparison to the values obtained for the condition without L-proline and N-PPG (asterisks, ANOVA with Bonferroni post hoc test) is indicated as ^#/^* *p* < 0.05; ^##/^** *p* < 0.01 and ^###/^*** *p* < 0.001. ns = not significant

The ability of L-proline to slow down the ATP loss and to prevent the cell toxicity observed during an 8 h incubation of astrocytes that had been treated with etomoxir plus UK5099 (Figs. 6 and 7) was abolished in the presence of the ProDH inhibitor N-PPG (Fig. 7), confirming that ProDH-mediated oxidation of L-proline is required to bypass the adverse effects of etomoxir plus UK5099 on starved astrocytes.

## Discussion

L-Proline is well known as energy substrate in yeast [13], protozoan parasites [14] and flying insects [6, 7, 48] and has also been discussed as potential energy substrate for mammals and mammalian cells, at least if the supply of other energy substrates is limited [49, 50]. We have recently shown that L-proline can be used by starved astrocytes to maintain a high specific ATP level [10]. To elucidate this process in more detail we have now studied the astrocytic consumption of proline both in the absence and the presence of other energy substrates.

In the absence of other energy substrates, L-proline is consumed by cultured astrocytes and maintains a high cellular ATP content, at least as long as the applied L-proline has not been completely consumed. This confirms recently published data [10]. Analysis of the concentration-dependency of L-proline consumption revealed a saturable process with half-maximal velocity calculated for an initial L-proline concentration of around 320 µM and a maximal L-proline consumption rate of around 100 nmol/(h x mg). Limiting for the astrocytic L-proline consumption and responsible for the saturable consumption rate could be either the uptake and/or the cellular metabolism of L-proline. The astrocytic consumption of L-proline was for all initial concentrations applied almost linear with time down to extracellular L-proline concentrations much lower than the calculated K_M_ value for the consumption. This suggests that mainly the cellular L-proline metabolism and not its uptake into the cells limits the L-proline consumption. This view is supported by the observations that the application of various alternative energy substrates, that are not structurally related to proline and are therefore unlikely to compete for L-proline uptake, lowered L-proline consumption, while inhibition of the mitochondrial metabolism of endogenous substrates such as pyruvate and fatty acids accelerated L-proline consumption.

L-Proline consumption demonstrates that astrocytes have the potential to take up extracellular L-proline. A broad range of transporters has been connected with L-proline uptake into mammalian brain cells [51, 52] As the expression pattern of some of these transporters in individual brain cells types such as astrocytes remains unknown [21], L-proline consumption by cultured astrocytes may be initiated by uptake via different transporters.

The K_M_ value of L-proline consumption (320 µM) is in the range of L-proline concentrations reported for serum (170 µM [53]; 450 µM [54]; 81–335 µM [55], 274 µM [56]; 51–271 µM [16]), suggesting that astrocytes in brain may encounter L-proline in concentrations that would allow substantial use of this amino acid for oxidative metabolism. Compared to serum, the L-proline concentrations in the cerebrospinal fluid are orders of magnitude lower with reported values in the low micromolar concentration range (6 µM [53]; up to 6 µM [57]; 1.35 µM [58]), suggesting that brain cells indeed consume substantial amounts of L-proline that is entering the brain from the blood vessels. Further studies are now required to elucidate whether substantial amounts of blood-derived L-proline are indeed taken up and consumed as energy substrate by the brain under physiological and/or pathological conditions, for example by measuring the arterio-venous difference in the L-proline concentrations of the blood that is entering and leaving the brain.

In contrast to L-proline, its stereoisomer D-proline is not consumed and not used as substrate to prevent ATP loss in glucose-deprived astrocytes. The two amino acid transporters PAT1 (SLC36A1) and PAT2 (SLC36A2) that have been described to mediate the transport of D-proline [59] appear not to be expressed in brain [60–62] or astrocytes [63–65] and also mammalian ProDH activity was found unaffected by the presence of high concentrations of D-proline, indicating the specificity of this ProDH for L-proline [66, 67]. Thus, the likely absence of a suitable transporter and the stereo-specificity of ProDH for L-proline explains why D-proline is not consumed by cultured astrocytes.

The ability of astrocytes to consume extracellular L-proline mainly depends on their potential to initiate catabolism of L-proline via oxidation by ProDH. When this enzyme is inhibited, both L-proline consumption by starved astrocytes and the maintenance of a high ATP content are prevented [10], demonstrating that L-proline consumption is almost exclusively initiated by the ProDH-mediated oxidation of L-proline in mitochondria. Several compounds have been reported to inhibit ProDH activity, including THFA [10, 40, 41], 5-Oxo [42] and N-PPG [42–45]. Analysis of the potential of these different known inhibitors of ProDH on the L-proline consumption by starved astrocytes revealed that N-PPG had a much higher potential to inhibit L-proline consumption with a half-maximal inhibition found in the micromolar concentration range than the other two inhibitors which had to be applied in millimolar concentrations to substantially affect L-proline consumption. A stronger inhibitory potential of N-PPG compared to the other inhibitors has also been reported for the ProDH of a human breast cancer cell line [42]. The structural L-proline analogues THFA and 5-Oxo are competitive inhibitors of ProDH [40, 42] that may not only compete with L-proline for binding at ProDH but also for uptake into astrocytes. In contrast, the structurally not related N-PPG is an irreversible ProDH inhibitor that inhibits ProDH activity by covalent modification of the enzyme-bound FAD [42–45]. As N-PPG is an irreversible inhibitor of ProDH that is effectively inhibiting L-proline consumption in much lower concentrations than THFA and as it remains unclear whether the structural analogue THFA may also compete for uptake with L-proline into astrocytes, only N-PPG was used for our further studies to inhibit L-proline consumption in astrocytes.

During incubation in the absence of any energy substrates, cultured astrocytes maintained a high ATP content for at least 8 h and toxicity was not observed for at least 24 h, while a longer starvation caused a decline in cellular ATP content that is followed by severe toxicity. These observations are consistent with literature data [10, 68] and confirm that astrocytes remain viable during starvation as long as the cellular ATP content does not decline below a threshold value of around 30% of the normal ATP content [68, 69]. The ability of starved astrocytes to maintain a high ATP content for hours depends on the mobilization of endogenous energy stores such as glycogen and fatty acids, and on the subsequent mitochondrial ATP regeneration from endogenous substrates [68]. Impairing such processes by inhibiting mitochondrial uptake of pyruvate (by UK5099) and activated fatty acids (by etomoxir) caused a rapid decline in cellular ATP content and severely compromised cell viability (current report, [68]). However, these adverse effects of the inhibitors were prevented by the presence of L-proline, demonstrating that mitochondrial L-proline metabolism is independent of the pathways blocked by the inhibitors applied, as expected for a substrate that fuels mitochondrial metabolism directly via ProDH. The protective effect of L-proline to bypass the inhibition of the utilization of endogenous energy substrates in starved astrocytes was abolished by inhibition of ProDH, demonstrating that the oxidation of L-proline by ProDH which initiates mitochondrial L-proline catabolism is essential for the rescuing potential of L-proline in energy-deprived astrocytes.

The oxidation of L-proline via ProDH, P5CDH and glutamate dehydrogenase to α-ketoglutarate and subsequent oxidation of glutamate-derived α-ketoglutarate can generate up to 34 ATP per L-proline molecule [11, 12], explaining that L-proline is able to serve as a highly potent extracellular substrate for ATP maintenance in starved astrocytes [10]. The ability of 1 mM L-proline to postpone ATP loss and prevent toxicity in starved astrocytes was as good as that of 1 mM glucose and both exogenous substrates showed additive effects, confirming the ability of astrocytes to efficiently use L-proline as energy substrate.

As the consumption of L-proline for the synthesis of other amino acids depends on the initial ProDH oxidation, L-proline catabolism is regulated by modulation of ProDH activity by downstream metabolite(s) that signal the availability of sufficient alternative energy substrates or the need for L-proline-derived metabolites in astrocytes. Such an inhibition of L-proline catabolism may spare L-proline for other purposes such as protein synthesis and limit the formation of glutamate in L-proline-exposed astrocytes.

L-Proline consumption by astrocytes was lowered in the presence of glucose or of other energy substrates. Such an inhibition of L-proline metabolism has previously been shown for pig enterocytes [70], colorectal cancer cells [49] and *Trypanosoma* [14]. The lowered L-proline consumption in glucose-fed astrocytes is unlikely to be caused directly by the presence of glucose, as the inhibition of L-proline consumption was maintained during the incubation even after the initially applied glucose had been completely metabolized by the cells. Thus, an accumulating glucose-derived metabolite is likely to impair L-proline consumption in astrocytes. This metabolite is most likely lactate that accumulates in glucose-fed astrocytes [71] and has been reported to inhibit ProDH in millimolar concentrations at least in liver [72] and in enterocytes [70].

Presence of pyruvate and lactate lowered L-proline consumption in astrocytes in a concentration-dependent manner, but the inhibitory potential of the applied lactate was higher than that of pyruvate. Most likely the lactate that is formed from applied pyruvate in astrocytes [35, 73] is inhibiting ProDH as pyruvate has been reported to have even in millimolar concentrations a lower inhibitor potential on ProDH than lactate [72]. This view is consistent with the presence of millimolar cytosolic concentrations of lactate in glucose-fed astrocytes, while cytosolic and extracellular pyruvate concentrations in glucose-fed astrocytes do not exceed the micromolar concentration range [71, 74]. Similarly, the observed inhibition of L-proline consumption in the presence of mannose is likely to be the consequence of the rapid production of lactate in mannose-fed astrocytes [71, 75]. The observed concentration-dependent inhibition of astrocytic L-proline consumption by a glucose-derived metabolite such as lactate could be quite relevant for the in vivo situation as it would allow astrocytes to adapt their oxidation of the alternative mitochondrial energy substrate L-proline on the availability of glucose, allowing the cells to upregulate L-proline catabolism to an appropriate extent that is needed to compensate for the limited glucose availability.

Partial inhibition of L-proline consumption in starved astrocytes was also found for incubations in the presence of acetate or β-hydroxybutyrate. These exogenous substrates are efficiently used as energy substrates by astrocytes [10, 35, 76], but cannot serve as source for the net formation of lactate, as their carbon is completely oxidized to CO_2_ in the citric acid cycle. Thus, an additional mechanism has to be assumed by which the presence of a mitochondrial energy substrate lowers the oxidation of L-proline. In millimolar concentration, succinate has been shown to have a substantially stronger inhibitory potential at least towards liver ProDH than other citric acid cycle intermediates such as α-ketoglutarate, fumarate and oxaloacetate [72] and has more recently been shown to inhibit ProDH by an uncompetitive mechanism [77]. The availability of mitochondrial succinate may also limit to some extend the basal L-proline consumption by glucose-deprived astrocytes, as L-proline consumption was found accelerated after inhibition of the mitochondrial catabolism of pyruvate and fatty acids that supply carbon for the citric acid cycle and thereby are likely to maintain a higher succinate level in mitochondria.

L-Proline has recently been shown to be used by astrocytes as efficiently as fatty acids as exogenous energy substrate for the maintenance of a high ATP content in starved astrocytes [10]. The rate of L-proline consumption is severely lowered in the presence of other energy substrates, which spares L-proline for the use as substrate for other processes, but also limits the formation of glutamine and glutamate that has been reported to be accelerated in L-proline-treated glucose-fed astrocytes [30]. Further studies are now required to investigate in more detail the interplay between glucose and L-proline metabolism concerning the utilization of L-proline for ATP regeneration and/or for the formation of L-proline-derived amino acids such as glutamate, glutamine, ornithine and arginine.

Disturbances of L-proline metabolism in the brain have been connected with neurological and psychiatric disorders [9, 16, 20, 21, 78]. Elevated L-proline concentrations in brain can be neurotoxic as they induce excitotoxicity and oxidative stress [21]. It remains to be elucidated, whether in addition to astrocytes other types of brain cells have the potential to metabolize L-proline as energy substrate, whether and to which extent astrocytes are involved in the L-proline metabolism in the healthy brain, whether the astrocytic contribution to brain´s L-proline metabolism is affected in disorders and whether astrocytic L-proline metabolism may help to lower the neurotoxic potential of elevated L-proline concentrations.

## Data Availability

Enquieries on original data should be directed to the corresponding author.
